# Pittsburgh compound B positron emission tomography detects cardiomyopathy in hereditary transthyretin amyloidosis patients with negative bone scintigraphy: a pilot study

**DOI:** 10.3389/fnume.2026.1747625

**Published:** 2026-02-09

**Authors:** Hendrea S. A. Tingen, Gilles N. Stormezand, Paul van Snick, Yingqi Hu, Paul Van Der Zwaag, Marish Oerlemans, Peter van der Meer, Bouke P. C. Hazenberg, Gert Luurtsema, Hans L. A. Nienhuis, Riemer H. J. A. Slart

**Affiliations:** 1 Department of Nuclear Medicine and Molecular Imaging, Groningen Amyloidosis Centre of Expertise, Groningen University, University Medical Centre Groningen, Groningen, Netherlands; 2 Department of Clinical Genetics, Groningen Amyloidosis Centre of Expertise, Groningen University, University Medical Centre Groningen, Groningen, Netherlands; 3 Department of Cardiology, University Medical Center Utrecht, Utrecht, Netherlands; 4 Member of the European Reference Network for Rare, Low Prevalence and Complex Diseases of the Heart: ERN GUARD-Heart' (ERN GUARDHEART); 5 Department of Cardiology, Groningen Amyloidosis Centre of Expertise, Groningen University, University Medical Centre Groningen, Groningen, Netherlands; 6 Department of Rheumatology & Clinical Immunology, Groningen Amyloidosis Centre of Expertise, Groningen University, University Medical Centre Groningen, Groningen, Netherlands; 7 Department of Internal Medicine, Groningen Amyloidosis Centre of Expertise, Groningen University, University Medical Centre Groningen, Groningen, Netherlands; 8 Biomedical Photonic Imaging Group, Faculty of Science and Technology, University of Twente, Enschede, Netherlands

**Keywords:** [^11^C]PiB, ATTR-CM, ATTRv, diagnosis, PET, type B fibrils, bone scintigraphy

## Abstract

**Purpose:**

To evaluate the effectiveness of positron emission tomography (PET) with [^11^C]-Pittsburgh Compound-B ([^11^C]PiB) for detecting transthyretin amyloid (ATTR) cardiomyopathy in patients with transthyretin gene (*TTR*) variants associated with reduced bone scintigraphy sensitivity, and its ability to detect brain involvement in hereditary transthyretin amyloidosis.

**Methods:**

This prospective case series included four hereditary ATTR amyloidosis patients with *TTR* variants associated with reduced bone scintigraphy sensitivity, and two patients with positive bone scintigraphy (one hereditary ATTR and one wild-type ATTR amyloidosis patient). All patients underwent diagnostic work-up at the Groningen Amyloidosis Centre of Expertise between April 2024 and March 2025, including [^11^C]PiB PET/CT. Cardiac and brain [^11^C]PiB uptake were assessed visually. Target-to-background ratios (TBRs) and cortical SUV ratios were calculated. TBR ≥1.09 was considered positive for ATTR cardiomyopathy and SUV ratios ≥0.7 was considered positive for brain involvement.

**Results:**

Cardiac [^11^C]PiB uptake was observed in three of four hereditary ATTR amyloidosis patients despite negative bone scintigraphy. In one of four patients, there was visually equivocal radiotracer uptake, but elevated TBRs did indicate ATTR cardiomyopathy. Conversely, both hereditary ATTR and wild-type ATTR amyloidosis patients with a positive bone scintigraphy had negative or inconclusive [^11^C]PiB PET results. Brain uptake was observed in two asymptomatic patients, while no uptake was seen in two patients with suspected brain involvement.

**Conclusion:**

[^11^C]PiB PET could be an effective tool for detecting ATTR cardiomyopathy in patients with *TTR* variants associated with reduced bone scintigraphy sensitivity. However, its utility for detecting brain involvement in symptomatic hereditary ATTR patients remains uncertain.

## Introduction

Transthyretin (ATTR) amyloidosis is a systemic disease characterized by deposition of misfolded transthyretin protein (1). It exists in two subtypes: wild-type ATTR (ATTRwt) amyloidosis, associated with aging, and hereditary ATTR (ATTRv) amyloidosis, caused by transthyretin gene (*TTR*) variants (2). While cardiomyopathy (ATTR-CM) is common in both subtypes, ATTRv amyloidosis can also affect the peripheral and central nervous system (1, 3).

ATTR-CM can be accurately diagnosed with bone scintigraphy, provided that immunoglobulin light chain amyloidosis has been excluded (4). Additionally, some centres use bone scintigraphy for cardiac screening in *TTR* variant carriers to detect subclinical ATTR-CM, an approach recommended in 2021 by the European Society of Cardiology and recently validated in a large multicentre cohort (5–7). However, bone scintigraphy has reduced sensitivity in certain *TTR* variants [such as p.(Tyr134Cys), early p.(Val50Met) and p.(p.Phe84Leu)], which complicates screening and may delay diagnosis of ATTR-CM in individuals carrying these variants (8–10). Despite negative bone scintigraphy findings, amyloid-binding PET tracers [^11^C]-Pittsburgh Compound B ([^11^C]PiB) and [^18^F]-flutemetamol have been shown to detect ATTR-CM in small cohorts of patients with these specific *TTR* variants (11–13), offering a potential non-invasive diagnostic modality for this group.

Furthermore, previous studies suggest that [^11^C]PiB and [^18^F]-flutemetamol can detect brain ATTR deposits in patients with these specific *TTR* variants (14–16). The advent of long-axial field-of-view (LAFOV) PET scanners has enabled simultaneous screening of both brain and heart and could provide a more comprehensive diagnostic approach to detect organ involvement in ATTRv amyloidosis, although short-axial field-of-view PET scanners can also be used.

This case series evaluates whether [^11^C]PiB LAFOV PET can detect heart and brain involvement in patients with *TTR* variants associated with reduced bone scintigraphy sensitivity.

## Methods

Four ATTRv amyloidosis patients with *TTR* variants associated with reduced bone scintigraphy sensitivity and two patients with positive bone scintigraphy results (one ATTRv and one ATTRwt amyloidosis) were included. All patients underwent diagnostic work-up for ATTR-CM at the Groningen Amyloidosis Centre of Expertise (GrACE) in the Netherlands, between April 2024 and March 2025. Data were collected from patient records, including symptoms, cardiac biomarkers, electrocardiography, echocardiography, [^99m^Tc]Tc-hydroxydiphosphonate bone scintigraphy and [^11^C]PiB PET/CT. Immunoglobulin light chain amyloidosis was excluded by blood and urine testing. The study was approved by the University Medical Centre Groningen ethical board (registration number: 17471).

Patients were scanned on a LAFOV PET/CT scanner (Biograph Vision Quadra, Siemens Healthineers, Knoxville) in a single bed position, capturing both heart and brain. A dynamic scan of 50 min was performed immediately after injection of 4–6 MBq/kg [^11^C]PIB (Reconstruction: [TrueX + TOF (UltraHD), 4 iterations, 5 subsets, 440 × 440 matrix, all-pass filtering.]. Attenuation correction was applied using a low-dose CT scan to correct for photon attenuation in the body. For analysis, images of the last ten minutes were summed, resulting in static images acquired 40 min post-injection.

Cardiac analysis was performed using Syngo.via version VB80D (Siemens Healthcare, Erlangen, Germany). Spherical volumes of interest (VOIs) were manually dilineated in the left atrial blood pool, interventricular septum and posterior and lateral walls of the left ventricle. The size of the VOIs depended on the size of the cardiac wall, with the cardiac wall volumes placed at the mid-ventricular level. Cardiac radiotracer uptake was assessed visually and scored as positive (+), negative (−) or equivocal (−/+). Additionally, target-to-background ratios (TBRs) were calculated by comparing myocardial SUV_mean_ to blood pool SUV_mean_. TBRs ≥1.09 were considered positive for ATTR-CM (17). For brain analysis, PET images were co-registered to anatomical magnetic resonance imaging (MRI) when available (*n* = 1), spatially normalized and segmented in grey and white matter regions employing PMOD version 4.4. Otherwise, PET images were spatially co-registered to a standard Montreal Neurological Institute template using elastic deformation. Predefined sets of VOIs, including the prefrontal cortex, orbitofrontal cortex, temporal cortex, anterior cingulate cortex and pons, were delineated using theHammers three-dimensional maximum probability atlas of the human brain (18). Cortical SUV ratio (SUVR) was calculated in standardized space by dividing the cortical SUV_mean_ by the pontine SUV_mean_ (16). Based on reported SUVRs in previous studies, SUVR ≥0.7 was considered abnormal (15, 16). Visual analysis by two nuclear medicine physicians identified cortical regions with specific uptake (+), nonspecific white matter uptake (−) or equivocal findings (−/+).

## Results

Patient characteristics are shown in Table 1. *TTR* variants included early onset p.(Val50Met) in two, p.(Tyr134Cys) in two and late onset p.(Val50Met) in one patient. All patients had suspected cardiac involvement based on increased wall thickness (≥12 mm) on echocardiography, conduction disorders and/or rhythm disturbances (19, 20). The delay between bone scintigraphy and the [^11^C]PiB PET/CT ranged from 2 to 19 months, with a median delay of 12.5 months. Two ATTRv amyloidosis patients had frequent transient focal neurological episodes (TFNE) characterized by aphasia, hemiparesis and hemisensory loss, indicating brain involvement. MRI confirmed leptomeningeal deposits in one patient and showed nonspecific abnormalities in the other, although elevated protein concentrations were found in the cerebrospinal fluid of this patient.

**Table 1:** Patient characteristics and cardiac evaluation for symptoms and signs of ATTR-CM.

Characteristics					Extracardiac disease manifestations			Treatment		Cardiac evaluation									
										HF	Biomarkers		ECG			TTE		BS	
Pt	Sex	Age at onset	Type	*TTR* variant	Neuropathy	Ocular	Brain	Time between start treatment and PET (months)	Drug		NT-proBNP (ng/L)	Hs-tropT (ng/L)	PM/ICD	AVB	IVCD	LVH	GLS	PS	H/WB
1	M	31	ATTRv	Early p.(Val50Met)	PNP, AN	+	−	35	Si	−	32	7	+	−	LAFB	−	−19.8%	0	n/d
2	F	65	ATTRv	Early p.(Val50Met)	PNP, AN	−	−	15	Si	−/+	412	31	+	n/a	IVCD	+	n/d	0	0.75
3	M	42	ATTRv	p.(Tyr134Cys)	PNP, AN	+	+	89	Si	−/+	209	21	−	−	−	+	n/d	0	0.60
4	F	39	ATTRv	p.(Tyr134Cys)	PNP, AN	+	+	37	Si	−/+	224	8	−	−	−	+	−18.2%	0	0.92
5	M	80	ATTRv	Late p.(Val50Met)	PNP	+	−	15	Si + St	+	181	16	−	−	−	+	−18.2%	1	4.50
6	M	81	ATTRwt	n/a	n/a	n/a	n/a	2	Si	−/+	3,386	44	−	2	−	+	−10.3%	3	n/d

ATTR-CM, transthyretin amyloid cardiomyopathy; Pt, patient; ATTRv, hereditary transthyretin amyloidosis; ATTRwt, wild-type transthyretin amyloidosis; n/a, not applicable; PNP, peripheral polyneuropathy; AN, autonomous neuropathy; Si, gene silencing therapy; St, transthyretin stabilizing therapy; HF, heart failure symptoms; NT-proBNP, N-terminal pro B-type natriuretic peptide; hs-tropT, high-sensitivity troponin T; ECG, electrocardiogram; PM, pacemaker; ICD, implantable cardioverter-defibrillator; AVB, atrioventricular block; IVCD, intraventricular conduction disorder; LAFB, center anterior fasicular block; TTE, transthoracic echocardiography; LVH, center ventricular hypertrophy; GLS, global longitudinal strain; n/d, not determinable; BS, bone scintigraphy; PS, Perugini score; H/WB, heart-to-whole body ratio.

**Figure 1:**
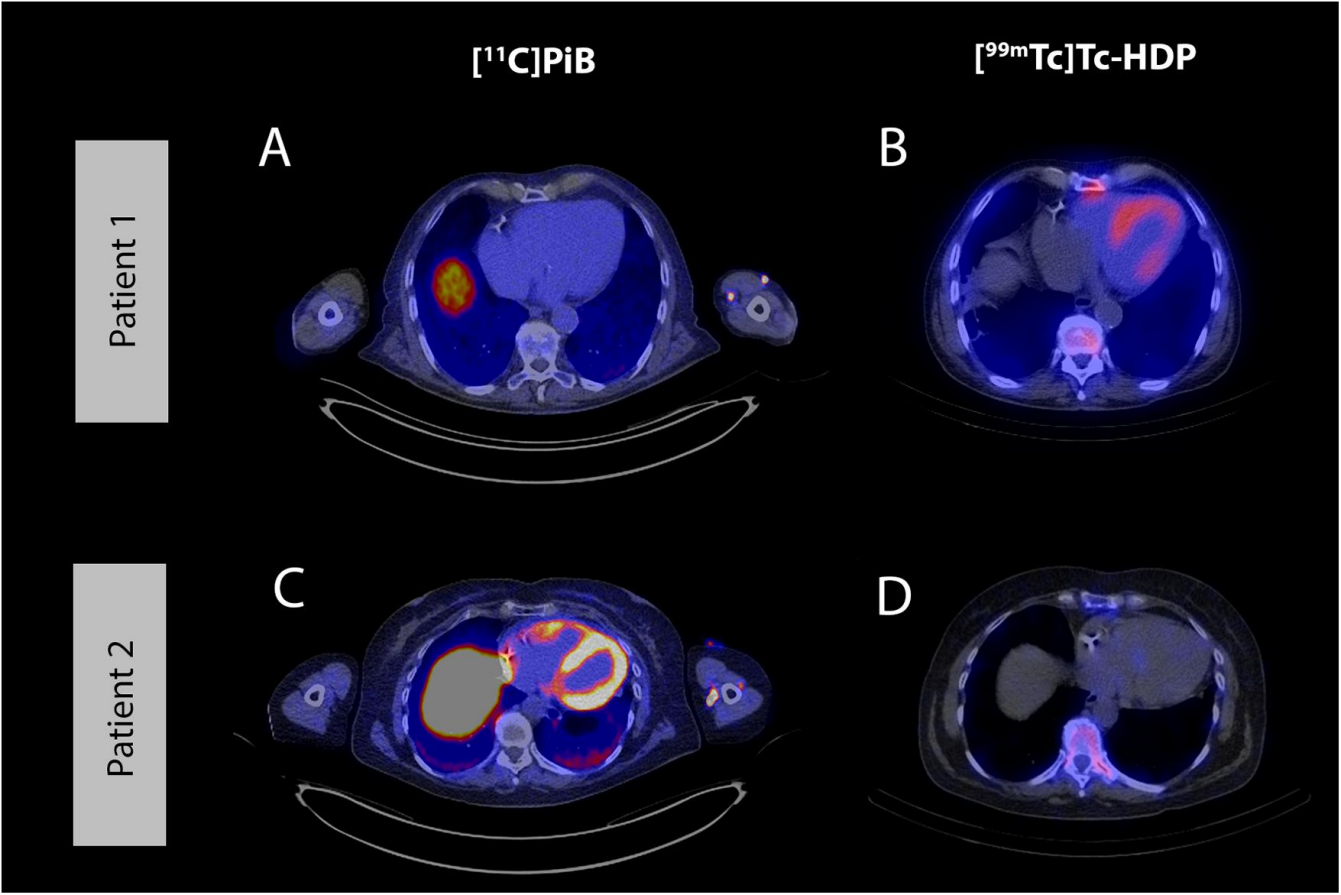
Example cases. Example of a negative [^11^C]PiB PET scan and positive [^99m^Tc]Tc-HDP SPECT/CT scan in an ATTRwt amyloidosis patient (A and B) and an example of a positive [^11^C]PiB PET scan and negative [^99m^Tc]Tc-HDP SPECT/CT scan in an ATTRv amyloidosis patient, carrying the early p.(Val50Met) variant (C and D). Axial [^11^C]PiB PET/CT images (A and C) and axial [^99m^Tc]Tc-HDP SPECT/CT images (B and D). [^11^C]PiB, Pittsburgh Compound B; PET, positron emission tomography; CT, computed tomography; [^99m^Tc]Tc-HDP, hydroxydiphosphonate; SPECT, single photon emission computed tomography; ATTRv, hereditary transthyretin amyloidosis.

Visual cardiac [^11^C]PiB uptake and increased TBRs were observed in patients with the early p.(Val50Met) variant and one patient with p.(Tyr134Cys) (Table 2 and Figure 1). In the other p.(Tyr134Cys) patient, visual cardiac uptake was equivocal, but lateral and posterior wall TBRs were increased. All patients with early p.(Val50Met) and p.(Tyr134Cys) variants had negative findings on bone scintigraphy. In contrast, the late p.(Val50Met) ATTRv amyloidosis patient and the ATTRwt amyloidosis patient both had positive findings on bone scintigraphy (Perugini grade 1 and 3), and no visual cardiac [^11^C]PiB uptake on PET. The posterior wall TBR was increased in the ATTRwt amyloidosis patient, while TBRs of the lateral wall and interventricular septum were below the cut-off, as were the TBRs of the late p.(Val50Met) patient.

**Table 2:** Findings on [^11^C]PiB PET-scan.

Pt	Type	Mutation	[^11^C]PiB PET									
			Cardiac				Brain					
			Visual	TBR septum	TBR lateral	TBR posterior	Visual	SUVR PFC	SUVR OFC	SUVR PC	SUVR TC	SUVR ACC
1	ATTRv	Early p.(Val50Met)	+	1.49	1.40	1.38	−	0.64	0.64	0.62	0.62	0.61
2	ATTRv	p.(Val50Met)	+	4.90	4.60	5.68	−/+	0.74	0.77	0.77	0.79	0.94
3	ATTRv	p.(Tyr134Cys)	−/+	0.75	1.38	1.19	−	0.59	0.61	0.58	0.59	0.63
4	ATTRv	p.(Tyr134Cys)	+	2.24	2.02	2.04	−	0.63	0.63	0.59	0.60	0.63
5	ATTRv	Late p.(Val50Met)	−	0.70	0.80	0.74	−	0.56	0.61	0.52	0.55	0.60
6	ATTRwt	n/a	−	1.07	0.97	1.24	−/+	0.76	0.79	0.64	0.63	0.96

[^11^C]PiB, Pittsburgh Compound B; PET, positron emission tomography; Pt, patient; ATTRv, hereditary transthyretin amyloidosis; ATTRwt, wild-type transthyretin amyloidosis; n/a, not applicable; TBR, target-to-background ratio; SUVR, retention ratio; PFC, prefrontal cortex; OFC, orbitofrontal cortex; PC, parietal cortex; TC, temporal cortex; ACC, anterior cingulate cortex. Bold values represent increased parameters.

Brain analysis revealed equivocal focal cortical [^11^C]PiB uptake in one asymptomatic early p.(Val50Met) patient and the ATTRwt amyloidosis patient. Three or more cortical regions in these patients had an increased SUVR, possibly indicating early brain amyloidosis. Although neurocognitive testing was not performed in these patients, they showed no clinical signs suggestive of cerebral or leptomeningeal involvement (no TFNEs, no evident cognitive impairment and no focal neurological deficits). No visual brain uptake was observed in the remaining patients, including the symptomatic patients, and all SUVRs were below the cut-off.

## Discussion

This case series suggests that [^11^C]PiB PET can detect ATTR-CM in patients with *TTR* variants associated with low bone scintigraphy sensitivity, such as early p.(Val50Met) and p.(Tyr134Cys), whereas [^11^C]PiB uptake was negative or inconclusive in patients with positive bone scintigraphy.

ATTR amyloid fibrils exist in two forms: type A fibrils, which are detectable by bone scintigraphy, and type B fibrils, which are associated with reduced sensitivity of bone scintigraphy (8–10). Previous evidence on the diagnostic performance of [^11^C]PiB PET for ATTR-CM has been inconsistent, likely due to differences in binding affinity between these fibril types (11, 12, 17, 21–24). Previous studies have shown higher cardiac [^11^C]PiB uptake in patients with type B fibrils than those with type A fibrils (11, 12), consistent with our findings. The previously reported negative results may explain why [^11^C]PiB PET has not yet been widely adopted as routine practice. Furthermore, its widespread implementation is constrained by limited availability, as it relies on the presence of an on-site cyclotron. However, our case series suggests that [^11^C]PiB PET could be an effective non-invasive diagnostic tool for detection of ATTR-CM in patients with *TTR* variants associated with low bone scintigraphy sensitivity. Since the use of [11C]PiB in ATTR-CM is investigational and not yet established, the results should be interpreted cautiously. Whether other amyloid PET tracers, such as [^18^F]-flutemetamol, [^18^F]-florbetapir and [^18^F]-florbetaben, offer similar diagnostic value in this population warrants further investigation, particularly given the wider availability of these tracers due to longer isotope half-lives. The same applies to the tracer currently under investigation, [^124^I]evuzamitide (25).

Despite having suspected ATTR-CM based on increased wall thickness, conduction disorders and/or rhythm disturbances (even requiring device implantation in 2 cases), the ATTRv patients with positive cardiac [^11^C]PiB PET had no evident heart failure symptoms and cardiac biomarkers were low. This atypical cardiac phenotype is consistent with prior findings in patients with type B fibrils and may contribute to diagnostic delays in this population (12, 26). [^11^C]PiB PET could enable early detection of ATTR-CM in this population, as it allows for non-invasive screening.

In the brain, two asymptomatic patients showed elevated cortical SUVRs, possibly indicating early amyloid deposition (16, 27). One of these had ATTRwt amyloidosis, in which brain involvement is not expected. The increased SUVRs may therefore reflect beta-amyloid deposits, indicating cerebral amyloid angiopathy or Alzheimer's disease, common diseases in the elderly (28–30). This is in line with positive amyloid PET findings in healthy controls, of which the likelihood increases with age (31). In contrast, two patients with frequent TFNEs, including one with MRI-confirmed leptomeningeal amyloid deposits, showed no elevated cortical [^11^C]PiB uptake. Our negative [^11^C]PiB PET findings contrast with previous studies, which reported positive PET findings in ATTRv amyloidosis patients with brain involvement (19, 21, 22). A possible explanation for our findings is that amyloid deposition may be confined to the leptomeninges, without affecting the brain parenchyma, as previously reported in ATTRv amyloidosis (16). Even with the improved resolution of LAFOV scanners, the resolution of PET is likely insufficient to detect such small anatomical structures (32).

Given the limited number of patients, this case series does not allow for definitive conclusions. However, it provides valuable feasibility data, demonstrating that [^11^C]PiB PET can be an effective non-invasive diagnostic tool for patients with *TTR* variants associated with low sensitivity of bone scintigraphy, a group currently lacking non-invasive diagnostic options. Another limitation is the analysis of static PET images instead of dynamic quantification, due to variation in scanning times within our cohort. However a previous study has shown that visual analysis and TBR calculation are reliable for detecting ATTR-CM (17). In addition, the TBR cutoff used to define cardiac [^11^C]PiB uptake was derived from a study using an earlier acquisition window (10–20 min), as no validated cutoff exists for imaging at 40 min post-injection and this may have led to an overestimation of PiB positivity. Additionally, as patients were already receiving treatment, this may have influenced PET results. Lastly, endomyocardial biopsies and cardiac MRI were not performed to confirm PET findings.

## Conclusion

[^11^C]PiB may serve as a non-invasive alternative to bone scintigraphy for screening and diagnosis of ATTR-CM in ATTRv amyloidosis patients with *TTR* variants associated with reduced bone scintigraphy sensitivity, though further validation is needed. Notably, while increased brain [^11^C]PiB uptake was observed in two asymptomatic patients, [^11^C]PiB PET failed to detect brain involvement in two symptomatic ATTRv amyloidosis patients, highlighting the need for further research.

## Data Availability

The raw data supporting the conclusions of this article will be made available by the authors, without undue reservation.
